# Ecological niche modelling and climate change in two species groups of huntsman spider genus *Eusparassus* in the Western Palearctic

**DOI:** 10.1038/s41598-022-08145-9

**Published:** 2022-03-09

**Authors:** Majid Moradmand, Masoud Yousefi

**Affiliations:** 1 Department of Plant and Animal Biology, Faculty of Biological Science and Technology, University of Isfahan, Isfahan, Iran; 2 Environmental Research Institute, University of Isfahan, Isfahan, Iran; 3 Stiftung Neanderthal Museum, 40822 Mettmann, Germany

**Keywords:** Ecology, Environmental sciences

## Abstract

The huntsman spiders’ genus *Eusparassus* are apex arthropod predators in desert ecosystems of the Afrotropical and Palearctic ecoregions. The *Eusparassus dufouri* and *E. walckenaeri* clades are two distinct taxonomic, phylogenetic, and geographic units concerning morphology, molecular phylogeny, and spatial data; but little is known about their ecological niche. We applied the maximum-entropy approach and modelled ecologic niches of these two phylogenetically closely related clades. Ecological niches of the two clades were compared using identity and background tests and two different metrics, the Schooner’s D and Warren’s I. We also predicted the impacts of climate change on the distribution of the two clades. The results of the identity test showed that the ecological niches of the two clades were different in geographic space but were similar in environmental space. While results of the background test revealed that the ecological niches of the two clades were similar in geographic and environmental space. This indicated that “niche conservatism” had an important role over the evolutionary time of allopatric diversification. However, the normalized difference vegetation index vs. topographic heterogeneity had influenced the niches of the *dufouri* and *walckenaeri* clades, respectively. The analyses recovered that the two clades’ climatically suitable habitats will increase under future climate (the year 2070). However, since the two clades are characterized by the narrow range of environmental optimum and the accordingly high limits of tolerance, they are vulnerable to climate change.

## Introduction

Spiders, order Araneae, are the main arthropod predators in the terrestrial ecosystems^1^. Spiders with nearly 50,000 species in 130 families are one of the most diverse groups of metazoan^2^. These venomous arachnids are proposed to be used for pest control in agroecosystems^3^. The spider venom and silk are subject of composition studies^4^ and application in pharmaceutical^5^, and technology of biomaterial^6^, respectively.

The Huntsman spiders, family Sparassidae, are large ambush predators partitioned in various habitats from humid rain forests to arid sand deserts. The family currently comprises 1290 described species in 89 genera^2^. The Stone huntsman spiders, genus *Eusparassus* Simon, 1903, composed of the largest predatory spiders (leg-span up to 14 cm) inhabiting xeric and subxeric regions of Africa, Mediterranean Europe via the Middle East to Central Asia^7,8^. Like other spiders, *Eusparssus* species are generalist predators but they mainly prey on insects and are able to subdue small vertebrates, indicating them as potential apex arthropod predators in the desert ecosystems^8,9^. *Eusparassus* represents one of the successful taxa from evolutionary perspective since it exists from at least 50 million years ago in the early Tertiary according to evidence of the amber fossil in Europe^10^. The genus currently comprises 30 species, majority of them are classified into six morphologic species-groups: the *Eusparassus doriae, dufouri*, *jaegeri, vestigator*, and *walckenaeri*^8^ while molecular phylogeny recovered and confirmed them as phylogenetically defined clades (exc. *vestigator;* no DNA data) based on four molecular markers^11^. The *dufouri* clade members are distributed in NW Africa and the Iberian Peninsula neighbouring the *walckenaeri* clade members which occur in Eastern Mediterranean, NE Africa, and the Arabian Peninsula. These two clades recovered as closely related taxa according to phylogenetic inferences^11^. The *Eusparassus* members and the entire family Sparassidae have never been the subject of any ecological niche modelling studies to date and our knowledge about factors that shaped their evolutionary history is scarce.

Every kind of living form has a range of specific environmental characteristics within which can have positive population growth; this range is called an ecological niche^12^. There are two kinds of ecological niches, the fundamental niche which is solely influenced by abiotic, and the realized one which is the result of interaction with biotic factors^13^. Species Distribution Modelling (SDM) methodologically uses a variety of statistical methods concerning large-scale climatic and topographic variables to estimate the ecological niche of taxa in relation to underlying environmental gradients^14^. These models are important tools in ecology, evolution and biogeography studies^15–18^. They have been used in studying niche evolution, niche dynamic, and niche comparisons in different taxonomic groups above and below species level^15,16,19–21^. Spiders have rarely been the subject of ecological niche modelling studies^22,23^. The previous SDM studies on spiders were geographically restricted to Europe and America devoting mainly to medically important biting spiders^24–27^, potentially model taxa to study biogeography and conservation biology^28–30^, and potentially endangered spiders^31–33^.

Here we applied the independent lines of evidence-based studies including morphology^7,8^ and molecular phylogeny^11^ integrated to geographical and environmental data to investigate the distribution modelling of two spider clades, *E. dufouri* clade (western Mediterranean Europe and Africa) and *E. walckenaeri* clade (eastern Mediterranean and partly in the Middle East and NE Africa), to test whether these two allopatric clades are different based on their ecological niches.

## Results

### Models performance

All niche models developed in this study performed well based on AUC metric (the *dufouri* clade’s ecological niche model: AUC = 0.976 ± 0.011, the *walckenaeri* clade’s ecological niche model: AUC = 0.982 ± 0.009, the *dufouri* clade’s climate niche model: AUC = 0.922 ± 0.016, and the *walckenaeri* clade’s climate niche model: AUC = 0.947 ± 0.021).

### Ecological niche model

**Figure 1:**
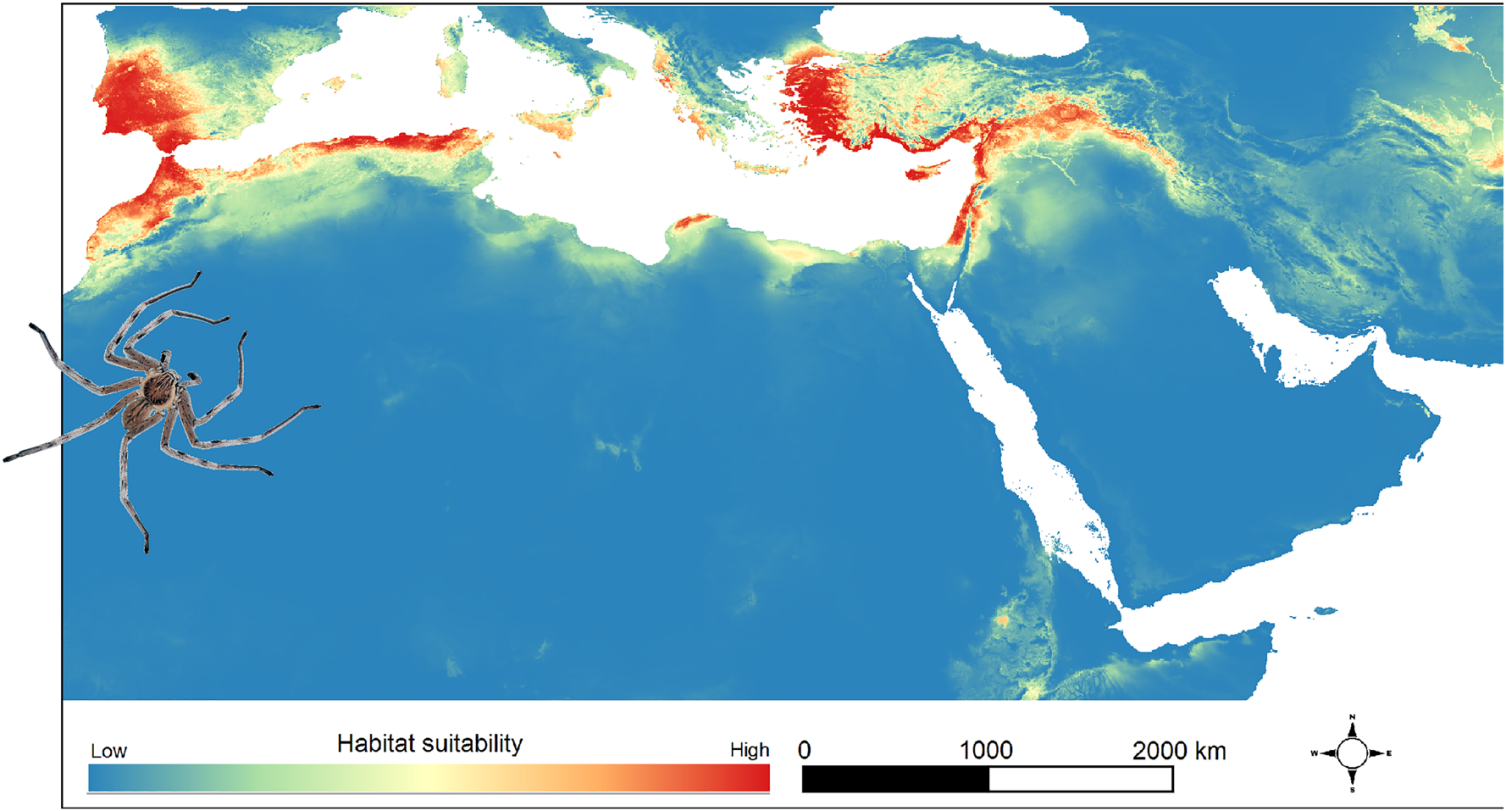
Distribution model of the *dufouri* clade. The distribution model was created using Maxent software 3.4.1 (https://biodiversityinformatics.amnh.org/open_source/maxent/) and mapped in QGIS 3.22.3 (https://www.qgis.org). The spider was photographed by Majid Moradmand.

**Figure 2:**
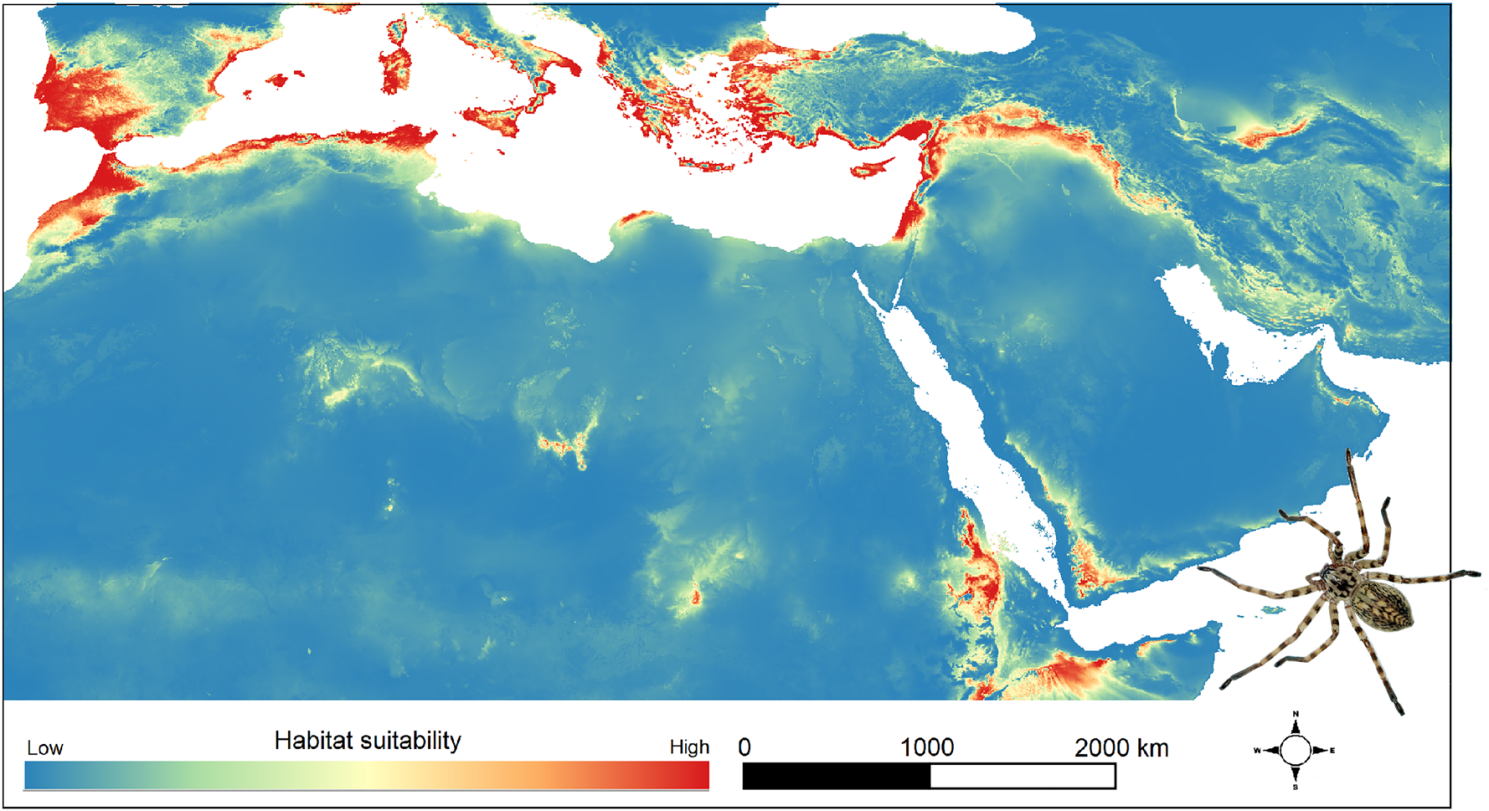
Distribution model of the *walckenaeri* clade. The distribution model was created using Maxent software 3.4.1 (http://biodiversityinformatics.amnh.org/open_source/maxent/) and mapped in QGIS 3.22.3 (https://www.qgis.org). The spider was photographed by Majid Moradmand.

The model predicted that the environmental condition of the present distribution range of the *dufouri* clade is suitable for the *walckenaeri* clade members and vice versa (Figs. 1, 2). The potential distribution range of the *walckenaeri* clade was estimated much more widely extending into those of the *dufouri clade* in NW Africa and Iberia*.* On the other hand, the environmental condition in the western Mediterranean (Iberian and NW Africa), where the *dufouri* clade is distributed, is potentially appropriate for the *walckenaeri* clade (Fig. 2). Moreover, the eastern Mediterranean (Greece and Turkey) had suitable environmental conditions for the *dufouri* clade as well (Fig. 1).

### Ecological niche across geographical and environmental spaces

**Figure 3:**
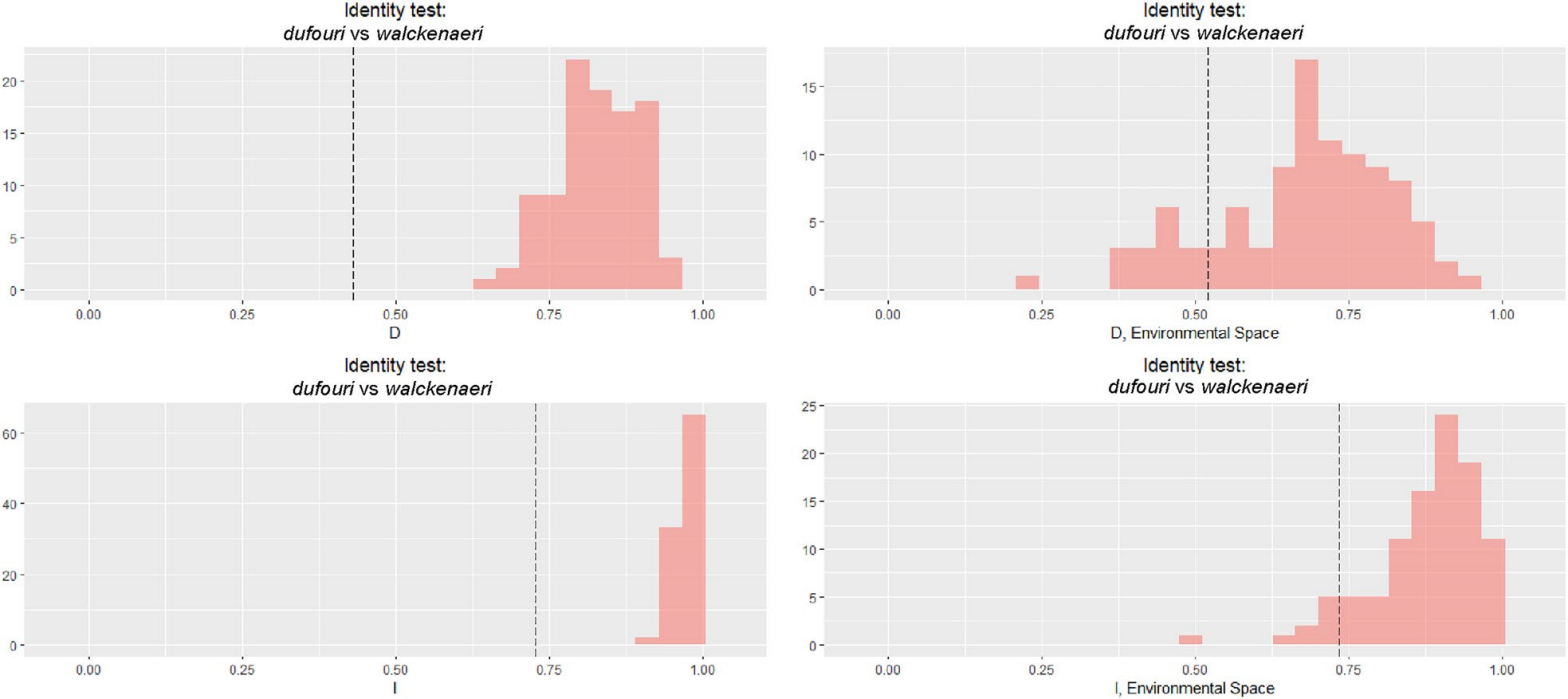
Results of identity test for the two clades (*dufouri* and *walckenaeri*) across the geographical and environmental spaces**.** Panels show histograms of 100 simulations of identity tests using two different metrics; the Schoener’s D and Warren’s I. Dotted lines indicate observed values. The figure is created using ENMTools 1.0.4 (https://github.com/danlwarren/ENMTools).

**Figure 4:**
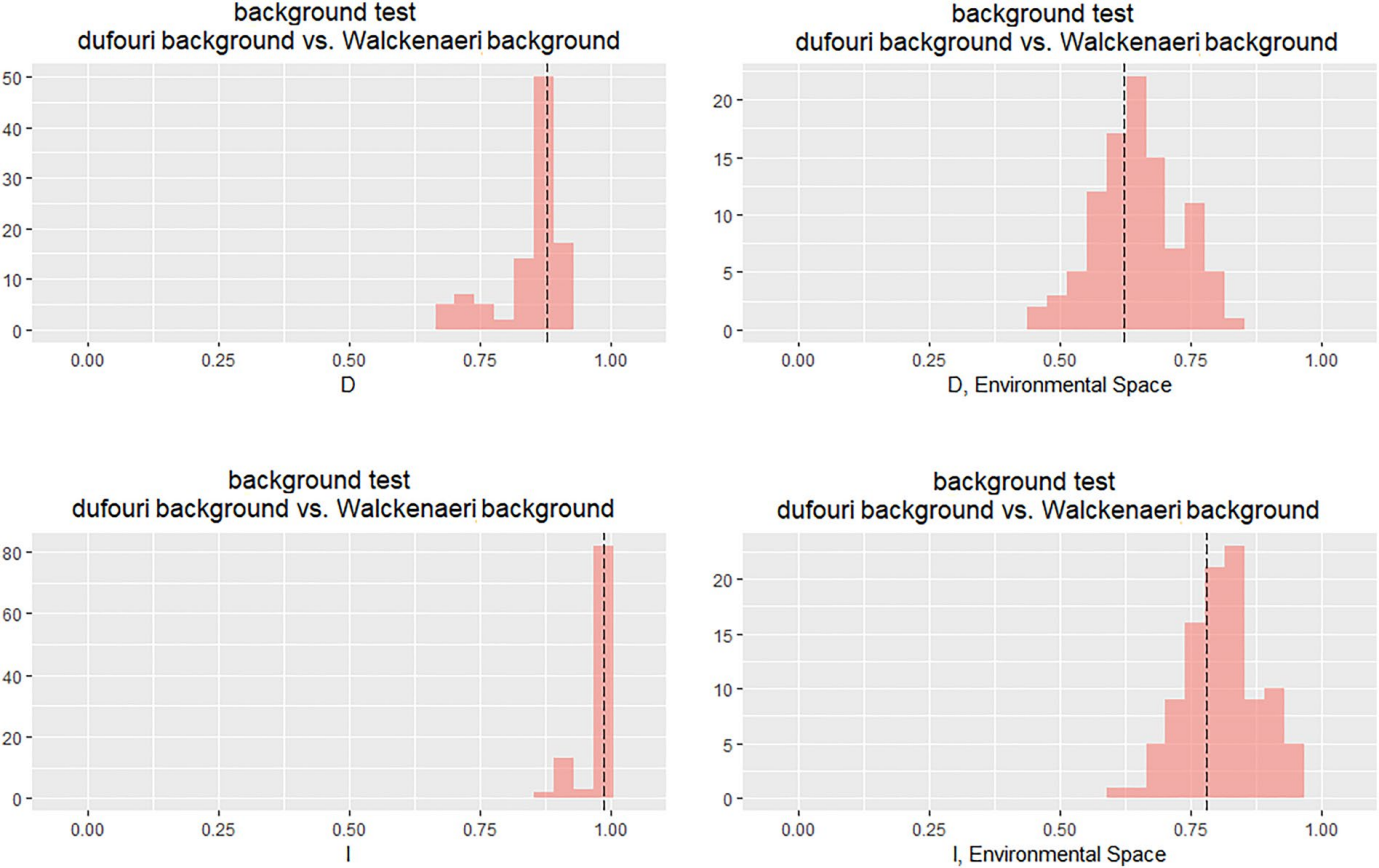
Results of background test for the two clades (*dufouri* and *walckenaeri*) across the geographical and environmental spaces**.** Panels show histograms of 100 simulations of background tests using two different metrics; the Schoener’s D and Warren’s I. Dotted lines indicate observed values. The figure is created using ENMTools 1.0.4 (https://github.com/danlwarren/ENMTools).

Comparing the ecological niche of the *dufouri* and *walckenaeri* clades using two identity tests, the Schoener’s D and Warren’s I showed that their niches are different only in geographical space but are similar in environmental space (Fig. 3). Results of two background tests, the Schoener’s D and Warren’s I showed that ecological niches of the *dufouri* and *walckenaeri* clades are similar in both geographical and environmental spaces (Fig. 4).

### Environmental variables and response curves

Results showed that the annual mean temperature (Bio1) with 44.9% contribution followed by the Normalized Difference Vegetation Index (NDVI) with 15.4% contribution were the most influential variables in shaping the ecological niche of the *dufouri* clade (Table 1). On the other side, for the *walckenaeri* clade, the Topographic Heterogeneity (TH) with 34% contribution and the annual mean temperature with 24.9% contribution were found to be the most important determinants of the ecological niche. Comparing the two clades, revealed that on one hand, the NDVI had much more influence on the *dufouri* clade (15.4%) than on the *walckenaeri* clade (3.6%); on the other hand, the TH had a greater impact on the *walckenaeri* clade (34%) than on the *dufouri* clade (7.9%). The response curves of the *dufouri* and the *walckenaeri* clades which were created using the environmental variables were similar and mostly unimodal (Fig. S1).

**Table 1:** Results of variable importance for the two clades (*dufouri* and *walckenaeri*).

Clades	Variables importance (%)					
	Bio1	Bio12	Elevation	TH	SRI	NDVI
*dufouri*	**44.9**	5.6	12.5	7.9	13.7	15.4
*walckenaeri*	24.9	14.4	17	**34**	6.1	3.6

Significant values are in bold.

*NDVI* normalized difference vegetation index, *TH* topographic heterogeneity, *SRI* solar radiation index.

### Climate change modelling

**Figure 5:**
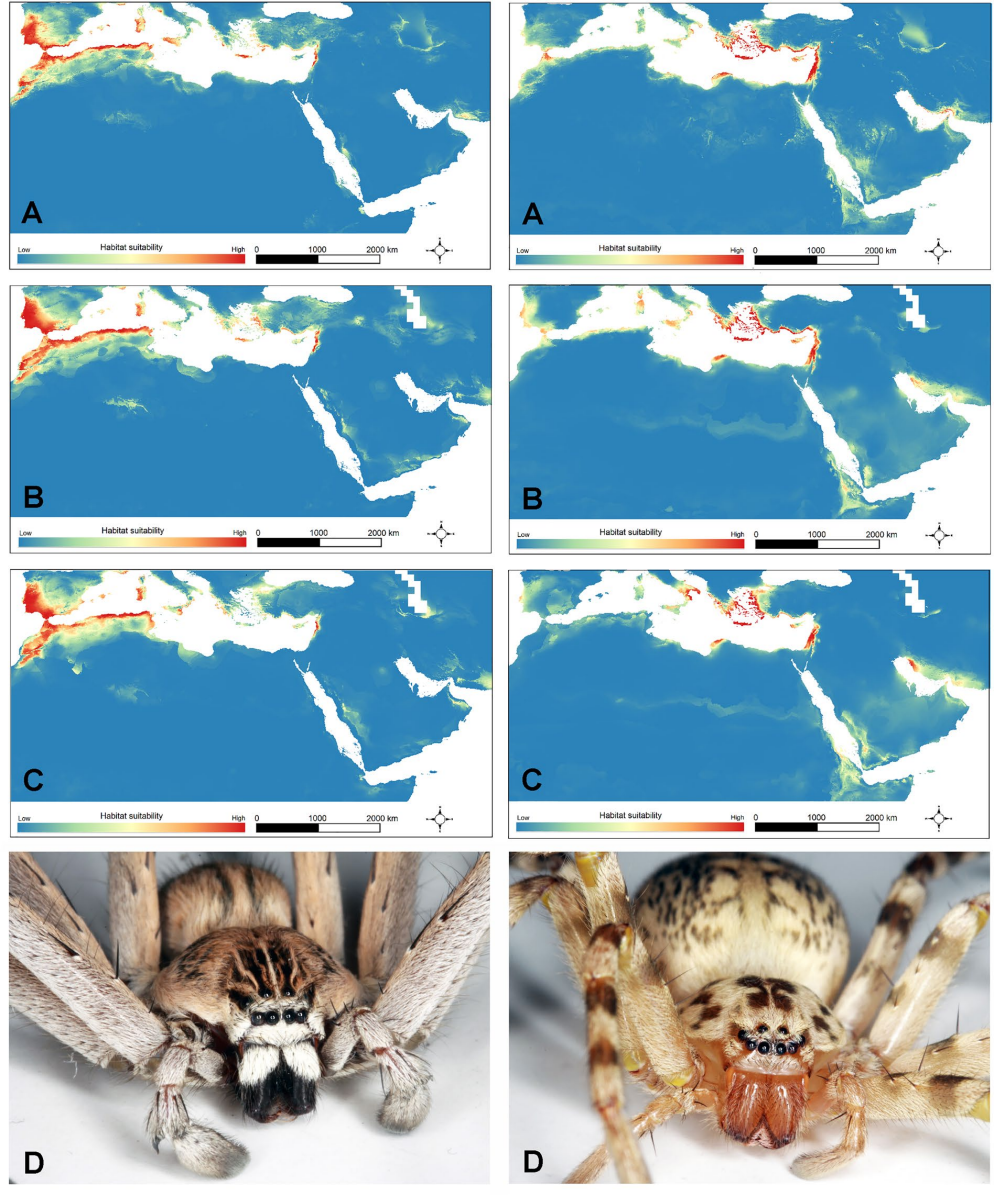
Climate suitability maps for the *dufouri* clade (left) and *walckenaeri* clade (right) for current (**A**) and future climate (2070), under CCSM4 RCP 8.5 (**B**) and MIROC5 RCP 8.5 (**C**), representatives of each clade, habitus from frontal view (**D**). The suitability maps were created using Maxent software 3.4.1 (http://biodiversityinformatics.amnh.org/open_source/maxent/) and mapped in QGIS 3.22.3 (https://www.qgis.org). The spiders were photographed by Majid Moradmand.

Results of predicting the impacts of climate change on the distribution of the two clades showed that climatically suitable areas will increase in the future (Fig. 5). Based on RCP 8.5 until 2070, climatically suitable habitats for the *dufouri* clade will increase between 16% (CCSM4) to 29% (MIROC5). Climatically suitable habitats for the *walckenaeri* clade will increase by 14% based on CCSM4 and while based on MIROC5 will remain stable under climate change (only 0.07% increase is expected). The response curves of the *dufouri* and the *walckenaeri* clades which were created using the bioclimatic variables were very similar and all unimodal (Fig. S2).

## Discussion

Despite being the most diverse group of organisms, but the terrestrial arthropods have rarely been used in the species distribution modelling studies compared to other animal phyla^27,34^, since the scarcity of available distribution data and fewer taxonomic studies^23^. Here, we estimated the potential distribution of the two clades of the spider genus *Eusparassus* in the Western Palearctic using ecological niche modelling. The two *Eusparassus* clades: *dufouri* and *walckenaeri,* are diagnosed by morphology, and distributed allopatrically in different geographic regions^8^, indeed molecular phylogeny recovered them as distinct monophyletic clades^11^. The result of the current study revealed that the environmental factors are not drivers of their distribution patterns and no significant niche differentiation was observed between these two clades.

The majority of the SDMs are built at the species level. But it is recommended that the taxonomic resolution below or above species level should be considered in distribution modelling^21,35,36^. Recently, applying other evolutionary taxonomic units to estimate spatial modelling has been developing but under different terms. For instance, the lineage-level based distribution models or LDM^37^ used intraspecific lineages of various species (e.g.^16,21,38^), and spatial distribution modelling at the Clade-level, applied data of inter-and intraspecific sampling for spatial modelling (e.g.^39^).

Most studies showed that climatic variables are the main driver of organism distribution and diversification^40^. The distributions pattern of spider communities is mainly influenced by the environmental variables (e.g.^41,42^), however, niche conservatism was observed in closely related spider taxa^26^. Studies on the subterranean spiders showed partial niche overlap between close relatives^33^. Investigation on spider diversification during evolutionary time with conservative climatic niche but allopatric distribution pattern is also reported in the spider family Ctenidae^30^. Ecologically similar but geographically different taxa are found in different groups of organisms too^39^ including insects^40,44,45^ and vertebrates^46,47^.

The diversification in two allopatric *Eusparassus* clades without divergence in the ecological niche is reported here. Both *Eusparassus* clades: *dufouri* and *walckenaeri* are recovered highly monophyletic and closely related lineage to each other and partitioned into two distinct geographic regions. There is no evidence of sympatric distribution of the species of these two clades except occasional overlap in their neighbouring borders^8^. Our result revealed that the geography and niche conservatism are the main drivers of diversification of these clades.

The responses of the *dufouri* and *walckenaeri* clades to environmental variables were generally similar. However, the impacts of environmental variables on the clades seem to be different between the two clades. Importantly, the impacts of NDVI vs. TH on the clades are correspondence with the field observation of the clade members in their natural habitats. Some species of the *dufouri* clade were collected from vegetation and trees showing foliage and arboreal habitat preferences of this group (see references^7,8^). In contrast, the *walckenaeri* clade members are well known as inhabitants of stony deserts where they mainly build their silken retreats under stones^8,9^. These pieces of evidence explain why NDVI and TH have significant impact differences in the *dufouri* and *walckenaeri* clades, respectively.

The topology of the respond curves to environmental and bioclimatic variables showed some levels of the narrow range in the optimum and accordingly high limits of tolerance. Any change in the optimal environmental condition can lead to a decrease in taxa performance and reach the zone of intolerance^48^. These data indicate that the clades are environmental specialists and sensitive to change in the corresponding environmental variables. We found out that the two clades’ climatically suitable habitats will increase under future climate (the year 2070) but because the two clades are characterized with a narrow range in the environmental optimum, we believe that they are vulnerable to climate change. The environmentally specialist spiders have poorly developed abilities of dispersal^49^. While aerial dispersal or ballooning is an efficient mechanism in spiders^1^, there is no evidence of this behaviour in Sparassidae, indicating their limited capability of dispersal compared to ballooning competitors. The lack of dispersal performance would cost the clade members by limiting them from reaching a suitable habitat. Consequently, the ongoing climate change would negatively affect the clades, and probably eliminate them from their habitats producing them as “climate change losers”. Since these spiders are the apex predators in their environment, thus planning conservation strategies are highly recommended.

## Methods

### Distribution data

**Figure 6:**
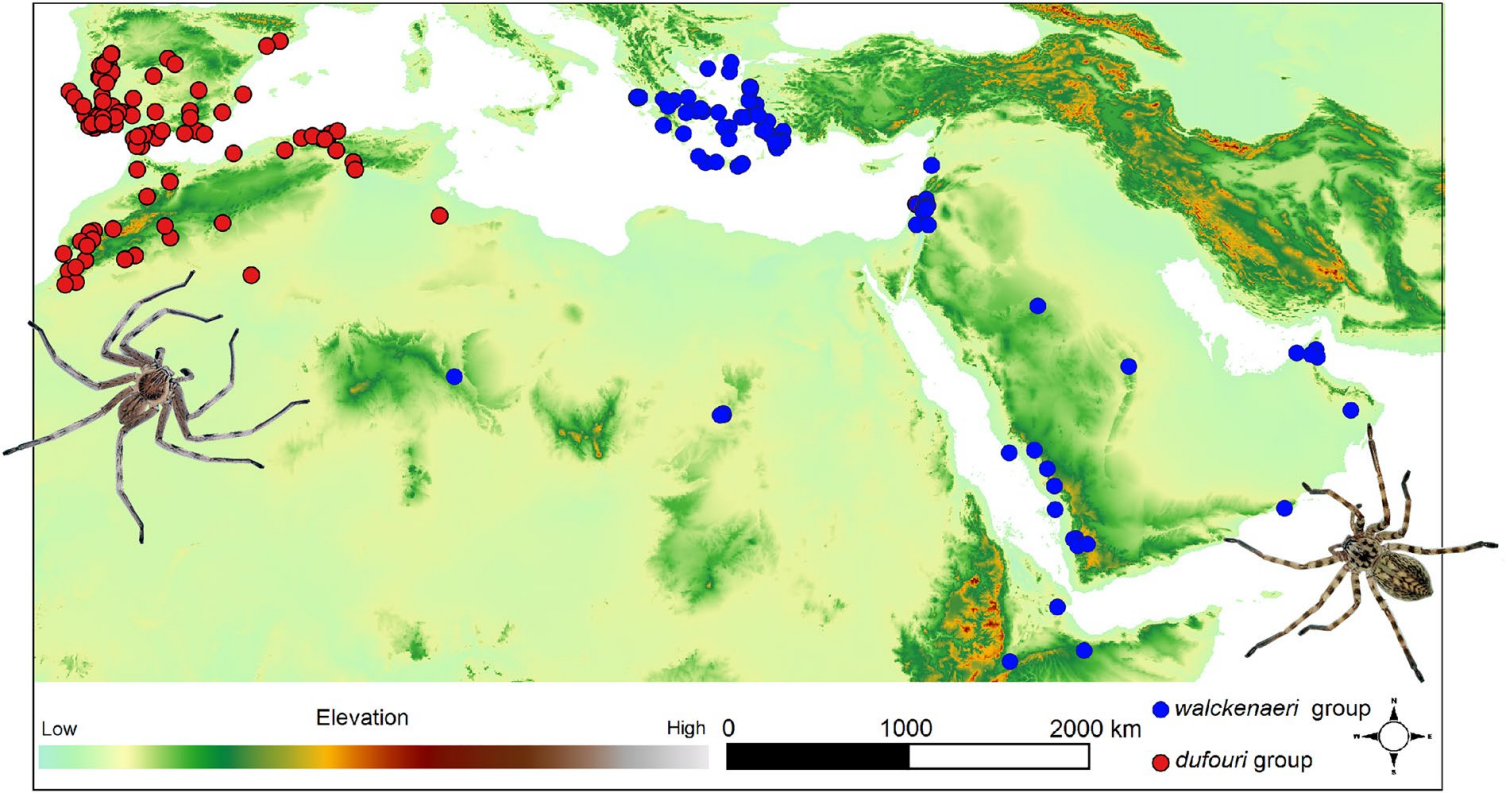
Distribution records of the *dufouri* clade and *walckenaeri* clade on a topographic overview of the study area. Elevation map was obtained from the Shuttle Radar Topography Mission (SRTM) elevation model (https://srtm.csi.cgiar.org/) and mapped in QGIS 3.22.3 (https://www.qgis.org). The spiders were photographed by Majid Moradmand.

Species distribution data of the *dufouri* and *walckenaeri* clades (three species per each, total N = 6 species) reconstructed after molecular phylogenetics^11^ were applied for georeferencing. The locality data of these species occurrences were obtained from different sources, partially by direct sampling in the field using GPS but mainly from museum collection labels and publications^7,8^ and GBIF source^50^. For the collection labels without coordination (containing just the exact geographical names), the online global gazetteers version 2.2 (http://www.fallingrain.com/world) was applied to assign longitude and latitude (georeferencing) to each locality. All forms of geographic coordinates were converted into decimal degrees. The data were used to build up an occurrence database of the studied taxa (Fig. 6). We carefully checked the clades data to clean the duplicate records. Because the environmental data were available at 5 km resolution, therefore we thinned the dataset to ensure that observations were at least 5 km apart. Overall, 133 distribution records were used in ecological niche modelling of *walckenaeri* clade and 111 occurrence records for *dufouri* clade.

### Environmental predictors

In this study, to develop an ecological niche model for each clade we used a combination of variables reflecting climate, topography, and plant productivity (Table 2). Climate variables (annual temperature (Bio1) and annual precipitation (Bio12)) and Solar Radiation Index at ~ 5 km spatial resolution were downloaded from WorldClim^51^. A digital elevation model(from the Shuttle Radar Topography Mission (SRTM) elevation model)^52^ of the study area was used to generate two topography variables; elevation and topographic heterogeneity (TH) in the raster package^53^. Then, the normalized difference vegetation index (NDVI) was used as an indicator of plant productivity, which may reflect resource availability. To avoid multicollinearity problems among variable predictors^54^, a variance inflection factor (VIF)^55^ analysis was run using the ‘usdm’ package^56^. We found low collinearity among the environmental variables (annual temperature = 6.977, annual precipitation = 2.554, elevation = 3.323, topographic heterogeneity = 1.636 and NDVI = 2.637).

**Table 2:** The list of environmental variables used for modelling *Eusparassus* clades in the current study.

Variable	Abbreviation	Units
Annual mean temperature	Bio1	Degrees Celsius
Annual precipitation	Bio12	Millimetres
Elevation	Elevation	Meters
Topographic heterogeneity	TH	Meters
Normalized difference vegetation index	NDVI	–
Solar radiation index	SRI	KJ m^−2^ day^−1^

### Ecological niche modelling and niche comparison

There are various statistical methods for SDM, but one of the most frequently applied methods is the maximum-entropy approach implemented in Maxent^57^. The efficacy of Maxent is proved to be useful in estimating the suitable environmental distribution range of the organisms such as spiders (e.g.,^24,28,29,58^). In this study, we used the maximum entropy approach^59^ to develop the ecological niche of the two phylogenetically clades (*dufouri* and *walckenaeri*) across their distribution range. For our models, maximum iterations were set to 500, convergence threshold was 0.0001, and 10,000 background locations were chosen from the entire study area (Fig. 5). Maxent output format was set Cloglog which gives an estimate between 0 and 1 of probability of presence. We applied the cross-validation method in Maxent and distribution points were randomly split into 10 folds containing an equal number of occurrences, and training models were created by eliminating each fold in turn^60^. Models performance was assessed using the Area Under the Curve (AUC) metric of the Receiving Operator Characteristic (ROC) curve^43,59,61^.

Ecological niches of the two clades were compared using identity and background tests and two different metrics, the Schoener’s *D*^62^ and Warren’s *I*^63^. Further, to understand whether niche similarity is smaller or larger than expected with respect to the clades’ geographical distribution we performed a background test using the Schoener’s *D*^62^ and Warren’s *I*^63^. We compared the two clades' ecological niches in geographic and environmental space separately^64^.

### Climate change assessment

We used the maximum entropy approach to predict the impacts of climate change on the two phylogenetically clades (*dufouri* and *walckenaeri*) across their distribution range. Two general circulation models of CCSM4 and MIROC5 for 2070 (average for 2061–2080) under the representative concentration pathways (RCP 8.5) were used. To predict the future distribution of the two clades the following nine bioclimatic variables were used: mean diurnal range (Bio2), temperature seasonality (Bio4), mean temperature of wettest quarter (Bio8), mean temperature of driest quarter (Bio9), precipitation of wettest month (Bio13), precipitation of driest month (Bio14), precipitation seasonality (Bio15), precipitation of warmest quarter (Bio18) and precipitation of coldest quarter for current (Bio19) and future climatic conditions. Bioclimatic variables for current and future climatic conditions were downloaded from Worldclim (https://www.worldclim.org/). In Maxent models, maximum iterations were set to 500, convergence threshold was 0.0001, and 10,000 background locations were chosen. To estimate the area of suitable habitats of each clade under current and future climatic conditions, we first converted continuous model outputs to presence/absence maps using the 10th percentile training presence threshold^35,65,66^. Then calculated area of suitable habitats under current and future climatic conditions using Raster package in R environment v. 3.4.3. Like ecological niche models for climate niche models, we applied the cross-validation method in Maxent and distribution points were randomly split into 10 folds containing an equal number of occurrences, and training models were created by eliminating each fold in turn^60^. Models' performance was assessed using the Area Under the Curve (AUC) metric of the Receiving Operator Characteristic (ROC) curve^43,59,61^.

## Supplementary Information

Supplementary Information.

## Data Availability

The datasets generated and analysed during the current study are available from described sources in the manuscript. Further information can be obtained from the corresponding author on reasonable request.
