# Supplementary material for: Ecological niche modelling and climate change in two species groups of huntsman spider genus Eusparassus in the Western Palearctic
Source: Sci Rep. 2022 Mar 9;12:4138. doi: 10.1038/s41598-022-08145-9 (PMC8907240; doi:10.1038/s41598-022-08145-9)
Supplement: Supplementary file 1 — Supplementary Information. [file 41598_2022_8145_MOESM1_ESM.pdf]

Supplementary Information

Ecological niche modelling and climate change in two species groups of huntsman spider genus *Eusparassus* in the Western Palearctic

M. Moradmand and M. Yousefi

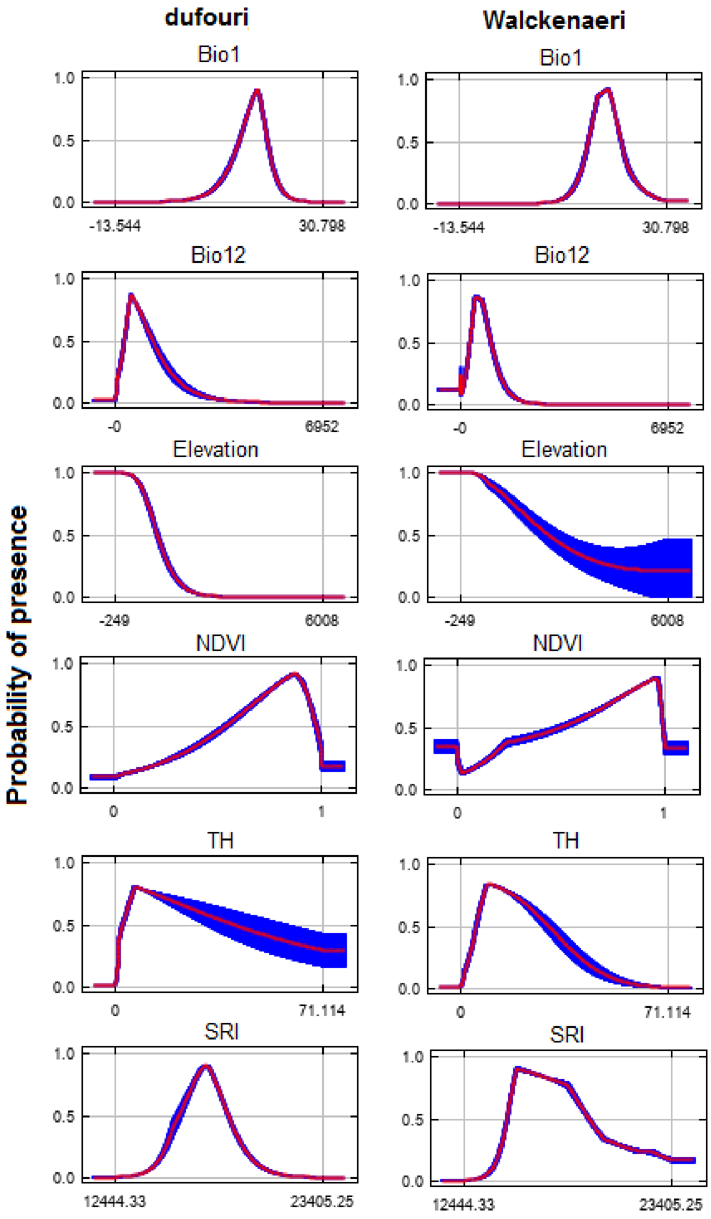

Figure S1. Response curves showing how each clade respond to environmental variables. Normalized Difference Vegetation Index (NDVI), Topographic Heterogeneity (TH), Solar Radiation Index (SRI).

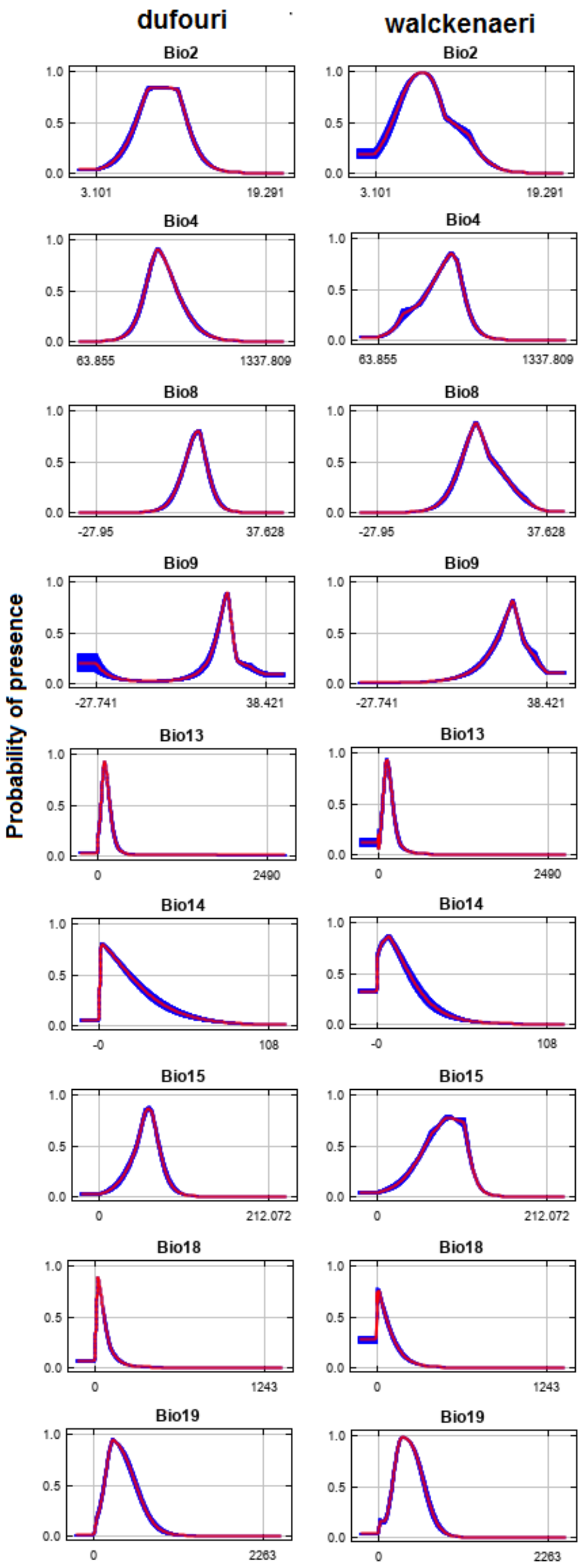

Figure S2. Response curves showing how each clade respond to bioclimatic variables.
